# Preoperative Chest Computed Tomography Screening Reduces the Perioperative Stroke Rate in Patients at Risk of Aortic Calcification

**DOI:** 10.3390/jpm14091005

**Published:** 2024-09-20

**Authors:** Tamer Ghazy, Maximillian Vondran, Marc Irqsusi, Martin Moscoso-Ludueña, Helmut Karl Lackner, Adrian Mahlmann, Ardawan J. Rastan

**Affiliations:** 1 Department of Cardiac and Thoracic-Vascular Surgery, University Hospital Gießen and Marburg, Philipps University of Marburg, 35043 Marburg, Germany; irqsusi@med.uni-marburg.de (M.I.); a.rastan@uk-gm.de (A.J.R.); 2 Department of Cardiac and Vascular Surgery, Klinikum Karlsburg, Heart and Diabetes Center Mecklenburg-Western Pommerania, 17495 Carlsburg, Germany; vondran.m@drguth.de; 3 Department of Cardiac and Vascular Surgery, Heart and Vascular Center Rotenburg, 36199 Rotenburg an der Fulda, Germany; m.moscoso-luduena@hkz-rotenburg.de; 4 Division of Physiology, Otto Loewi Research Center for Vascular Biology, Immunology and Inflammation, Medical University of Graz, 8010 Graz, Austria; helmut.lackner@medunigraz.at; 5 Centre for Vascular Medicine, Clinic of Angiology, St.-Josefs-Hospital, Katholische Krankenhaus Hagen gem. GmbH, 58099 Hagen, Germany; mahlmanna@kkh-hagen.de; 6 Department of Internal Medicine III, University Hospital Carl Gustav Carus, Technische Universität Dresden, 01307 Dresden, Germany

**Keywords:** computed tomography, perioperative management, aortic calcification, stroke, cardiac surgery

## Abstract

Objectives: We evaluated the effect of preoperative chest computed tomography (CT) screening on the perioperative stroke rate in cardiosurgical patients at risk of aortic calcification. Methods: Between May 2019 and April 2020, 129 patients at risk of aortic calcification underwent non-contrast chest CT screening before their procedure. They were assigned to Group 1 and compared with a historical Group 2, who were treated the previous year without a preoperative CT scan. The primary endpoint was to determine postoperative stroke occurrence. The secondary outcomes were the rate reintubation/tracheostomy, the length of hospital stay, and any change in surgical strategy based on the CT findings. Results: Groups 1 and 2 comprised 129 and 261 patients, respectively. Group 1 had a lower left ventricular ejection fraction, less carotid stenosis, a history of carotid endarterectomy, and a longer cross-clamp time. The surgical strategy was changed for 6 patients in Group 1. Group 1 had a significantly lower stroke rate. No significant differences were observed in reintubation and tracheostomy rates, or length of hospital stay. Lack of CT screening, age, aortic valve surgery, aortic surgery, and rethoracotomy were identified as independent risk factors for a stroke. Conclusions: Preoperative non-contrast chest CT screening of patients at risk of aortic calcification reduces postoperative stroke through adaptation of the surgical approach and should be used routinely in these patients.

## 1. Introduction

Postoperative stroke is a devastating complication of cardiac surgery, with an incidence ranging from 1.8–9.6% in the general adult cardiosurgical population [1], and up to 17% in patients aged >65 years undergoing aortic valve replacement [2]. Recent studies on patients with aortic valve stenosis (AS) showed a higher calcification load in high-risk patients, with two-thirds of patients having a calcium score of ≥1000 Hounsfield units (HUs), and a significant association exists between each 1000 HU increment increase and a higher all-cause mortality [3]. Meanwhile, several reports and meta-analyses confirmed significantly lower stroke rates in aortic no-touch coronary surgery than in standard coronary surgery with aortic clamping [4,5,6].

Previous investigations have identified calcification of the ascending aorta as the most important predictive factor for a postoperative stroke [7]. This concurs with research studies that reported aortic arch calcification as an independent risk factor for stroke [8,9,10]. Furthermore, in cases of ischemic stroke, aortic arch morphology may add a further challenge for emergency endovascular treatment and might indicate alternative approaches [11].

Although preoperative computed tomography (CT) has been a standard measure for catheter-based valve intervention for many years, it has not been adopted as a general standard for open cardiac surgery. CT imaging is a non-invasive method for the detection of calcifications and other pathologies in at-risk patients [12]. While unnecessary exposure to radiation, additional costs, and more complex planning might be reasons for non-adoption in some patients, CT imaging might be helpful in patients with a high risk of aortic calcification (AC) [12]. Unfortunately, the benefits of cross-sectional imaging have not been thoroughly investigated.

In May 2019, owing to discussions of our intensive heart team with patients scheduled for transcatheter aortic valve implantation (TAVI), and with the realization of the high prevalence of AC, we decided to expand our cardiosurgical preoperative diagnostics in patients with a high risk of AC undergoing open heart surgery, and established a structured protocol for preoperative AC detection using preoperative non-contrast CT imaging in those patients. A high risk of AC was presumed with any of the following criteria: age ≥ 75 years, peripheral vascular disease (PVD), carotid artery stenosis > 50%, aortic calcification on chest radiography, and chronic dialysis.

The aim of this study was to investigate the effect of routine non-contrast CT imaging of the thoracic aorta on perioperative stroke rates in patients at risk of AC. Furthermore, this study aimed to analyze the rate and mode of change in operative strategies based on CT findings.

## 2. Materials and Methods

In this retrospective case-control study, patients who underwent open cardiosurgical procedures between May 2019 and April 2020 in a single Center were screened for inclusion in this analysis. The inclusion criteria were:

- Age over 18 years
- Undergoing open heart surgery with aortic cross-clamping
- Having at least one of the following criteria
  ○ Age over 75 years
  ○ Peripheral vascular disease
  ○ Carotid artery stenosis > 50%
  ○ Aortic calcification on chest radiography (X-ray)
  ○ Chronic dialysis
- Undergoing preoperative non-contract CT imaging of the chest.

Patients who underwent emergency procedures and did not receive preoperative CT imaging due to the emergency nature of the procedure were excluded.

All patients who met the inclusion and exclusion criteria were included in the analysis as the experiment group (Group 1).

For the construction of the control group, all patients who underwent open heart surgery between May 2018 and April 2019 (i.e., the historical cohort, before the introduction of the CT screening protocol in our institution) were screened for the same inclusion and exclusion criteria as Group 1. The patients of the historical cohort who met the inclusion and exclusion criteria, except for undergoing a preoperative CT-scan, served as the control group (Group 2).

Preoperative, operative, and postoperative data were prospectively collected from the hospital database and retrospectively analyzed. The primary endpoint of the study was the incidence of a temporary or permanent early postoperative stroke. The secondary outcome parameters were any changes in surgical strategy based on the CT findings, reintubation and tracheostomy rates, and duration of hospital stay.

This study was approved by the local Ethics Committee (Giessen University Ethical Committee, decision number 205/23), and the requirement for informed consent was waived by the Institutional Review Board.

### Statistical Analysis

Continuous variables are presented as a mean ± standard deviation. Binary data are presented as percentages. Categorical variables were compared using the chi-squared and Fisher’s exact tests. Continuous variables were compared using Student’s *t*-test. Independent effects were studied using a stepwise logistic regression model. Statistical significance was set at *p* < 0.05 (two-sided), and all statistical analyses were performed using SPSS (version 27) software (International Business Machines Corp., Armonk, NY, USA).

## 3. Results

### 3.1. Patient Cohorts and Preoperative Data

A total of 390 patients (283 male) fulfilled the inclusion criteria. Groups 1 and 2 included 129 (96 male) and 261 (187 male) patients, respectively. Data analysis showed comparable study groups with no statistical differences in patient characteristics, such as age, diabetes, arterial hypertension, pulmonary hypertension, New York Heart Association (NYHA) functional classification, active smoking status, cardiogenic shock, recent resuscitation, atrial fibrillation, coronary artery disease, peripheral vascular disease, aortic aneurysm, creatinine level, chronic dialysis, and chronic obstructive lung disease. When compared against Group 2, patients in Group 1 had less carotid stenosis (18.6% vs. 28.0%, *p* = 0.047), a higher rate of recent infarctions (3.1% vs. 0.4%, *p* = 0.043), and higher left ventricular ejection fractions (55 ± 11 vs. 52 ± 14, *p* = 0.02). The groups had comparable histories of cerebrovascular disease (14% vs. 18%, *p* = 0.616). Detailed demographic data and preoperative risk factors are presented in Table 1.

### 3.2. Intraoperative Data

Urgent procedures were reported in 38% and 45% of patients in Groups 1 and 2, respectively, with no significant difference between the groups (*p* = 0.277). The analysis showed no significant difference in the surgical approach (median sternotomy versus a minimally invasive approach), or cross-clamp time (CBP) time. Group 1 showed longer operative and cardiopulmonary bypass times than Group 2 (268 ± 84 vs. 248 ± 95, *p* = 0.046; and 121 ± 67 vs. 107 ± 58, *p* = 0.032, respectively). The distribution of most procedures (valve surgery, coronary surgery, aortic surgery, atrial ablation, or combined procedures) did not differ significantly between groups (Table 2). However, patients in Group 1 underwent fewer carotid endarterectomy procedures than those patients in Group 2 (1.6% vs. 6.2%, *p* = 0.043).

Six patients in Group 1 were preoperatively determined to have massive aortic calcifications: two patients had severe aortic valve stenosis, and their open surgical procedure was switched to transcatheter aortic valve implantation. Four patients had coronary heart disease, three of whom underwent off-pump coronary bypass, and one of whom underwent on-pump beating-heart coronary bypass.

### 3.3. Postoperative Data

#### 3.3.1. Study Endpoints

Postoperatively, 3.1% of the patients in Group 1 met the primary endpoint. In Group 2, 8.4% of the patients met the primary endpoint. The analysis showed significant differences between the groups (*p* = 0.047). No between-group differences were observed in terms of postoperative reintubation/tracheostomy, or length of hospital stay (Table 3).

#### 3.3.2. Postoperative Stroke Data Analysis

**Figure 1:**
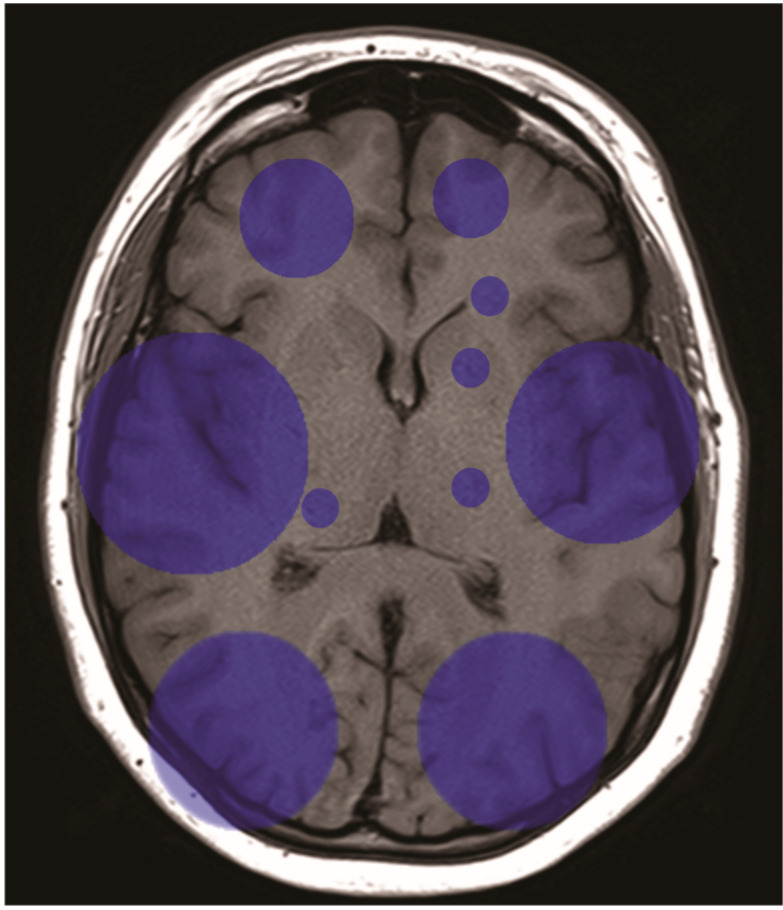
Distribution of detected cerebral infarction. Larger circles denote a higher incidence of infarction in that location.

An analysis of patients who suffered a postoperative stroke showed that all patients had ischemic stroke. No hemorrhagic cases were reported. The distribution of the infarction locations is shown in Figure 1. Infarctions were detected more often in the middle and posterior cerebral territories than in the anterior territories, with no significant differences between the two hemispheres. Of the 26 patients who had a postoperative stroke, 1 died, 1 was transferred to neurosurgery for decortication, 19 were transferred to neurological rehabilitation, 2 were transferred to geriatric rehabilitation, and 3 were transferred to cardiosurgical rehabilitation after the improvement of symptoms, with no significant difference between the groups (*p* = 0.783). There was no significant difference in the Rankin score improvement before discharge between those patients in whom a demarcated infarction was detected and those patients who did not show early CT demarcation (*p* = 0.265).

#### 3.3.3. Univariate Analysis of Risk Factors Affecting Postoperative Stroke

Univariate analysis identified a lack of CT screening, age, aortic surgery, aortic valve surgery, and rethoracotomy as risk factors for a postoperative stroke (Table 4).

Carotid stenosis and endarterectomy were not associated with higher postoperative stroke rates (*p* = 0.347 and *p* = 0.282, respectively).

#### 3.3.4. Multivariate Analysis of Risk Factors Affecting Postoperative Stroke

A reliable stepwise logistic regression model was achieved after three steps In the first step, the model was built to include age, sex, and BMI as model variables, and the model achieved a Nagelkerke R^2^ of 0.044 (i.e., an unreliable model). In the second step, CT screening was added as a variable, and the model achieved an R^2^ of 0.069 (i.e., still an unreliable model). In the third step, aortic valve surgery, aortic surgery, rethoracotomy, aortic aneurysm, and coronary surgery were added, and the model achieved a robust R^2^ of 0.218 (i.e., a reliable model). The logistic regression analysis of the regression model showed a significant independent effect of CT screening on postoperative stroke rate (*p* = 0.032, Table 5).

## 4. Discussion

Postoperative stroke is a frightening complication of cardiac surgery; it is the Achilles heel in studies comparing conventional cardiac surgery and interventional approaches to coronary artery and aortic valve disease [13,14,15].

Although multifactorial, aortic calcification remains an important factor affecting neurological outcomes in patients undergoing cardiac surgery [7]. Van der Linden et al. reported stroke rates of 1.8% in unselected patients undergoing cardiac surgery and 8.7% in patients with calcified aortas [7].

Epi-aortic ultrasound (US) and CT imaging are the most reliable methods of detecting aortic calcifications. US is an easy-to-use method that can detect calcifications and soft plaque in the ascending aorta, which can guide the surgical team to modify the cannulation and/or clamping site if calcifications are detected, and consequently reduce postoperative stroke rates [16,17]. Its use is recommended by the European Society of Cardiology (ESC) and the European Association for Cardiothoracic Surgery (EACTS) for the detection of atheromatous plaque and selection of the optimal surgical strategy [18]. However, it can only be performed intraoperatively after surgical exposure of the aorta. This might not be a major problem in patients undergoing multivessel coronary artery bypass grafting because the surgical strategy can be changed intraoperatively to aortic no-touch off-pump surgery. However, in patients who undergo valve surgery, the treatment strategy itself might have to be changed for cases of major aortic calcification, and should be decided before the patient undergoes surgery.

The ESC/EACTS guidelines on the diagnosis and treatment of aortic diseases recommend CT imaging for the detection of aortic calcification, as it is more capable than magnetic resonance imaging in this regard and provides better visualization of the entire thoracic aorta than ultrasound [19]. However, a CT scan exposes patients to a higher effective dose of radiation than standard chest radiography (7.8 vs. 0.02 mSv) [20], which adds to the financial burden and is logistically more demanding [19]. Therefore, CT angiography and non-contrast CT imaging are currently not standard in the preoperative preparation of patients undergoing cardiac surgery. Except for those patients whose procedures require preoperative CT imaging (e.g., aortic surgery or transcatheter aortic valve implantation), it is at the discretion of the cardiosurgical unit to decide when to include CT imaging in preoperative diagnostics. Nevertheless, a cohort study analyzing the data gathered during the COVID-19 pandemic, where all cardiosurgical patients underwent preoperative CT imaging, revealed incidental findings which led to a change in the operative decision-making in 7.2% of patients. The incidental findings included aortic calcifications, mitral annular calcifications, vascular anomalies, and pulmonary and mediastinal findings [21]. These results concur with our results and further call for a consensus stating the risk to groups in which a preoperative CT screening is warranted, and the benefit of the screening outweighs the financial and logistic burden. The ESC/EACTS guidelines for myocardial revascularization recommend preoperative CT imaging in older patients, or in patients with a higher probability of aortic calcification, to reduce the risk of a postoperative stroke (class of recommendation: IIa; level of evidence: C); however, there are no clear definitions of when a patient should be considered high-risk [18]. We hope that our study results will help form a consensus in this regard.

Whether CT imaging should be performed using a contrast agent remains debatable. A contrast agent enables surgical teams to make an optimal evaluation of the thoracic aorta to detect any concealed aneurysms, malformations, atheromata, thrombi, or penetrating aortic ulcers [19]. However, it is not required for the detection of calcifications, and can influence renal function, which may be detrimental in patients with preoperative renal impairment, especially if applied shortly before surgery [22]. We believe that contrast-agent CT imaging should not be a routine screening protocol and should be reserved for patients with a high suspicion of aortic pathology.

In a large cohort study, Iribarren et al. reported that age, smoking, and hypertension were independent risk factors associated with aortic calcification [23]. This was later confirmed by a multi-ethnic study of atherosclerosis [24]. Demer and Tintut described the pathobiology of aortic calcification and described atherosclerotic diseases and end-stage renal diseases as important independent factors associated with vascular calcification, two common comorbidities in cardiosurgical patients [25]. Lee et al. added new insights into its mechanism at the cellular and molecular levels [26] and helped explain the higher incidence of aortic calcifications in patients with the above-mentioned comorbidities. With increasing age and comorbidities in cardiosurgical patients, as shown in large database analyses [27], it is expected that a higher number of patients will develop aortic calcifications, necessitating a more effective detection strategy.

**Figure 2:**
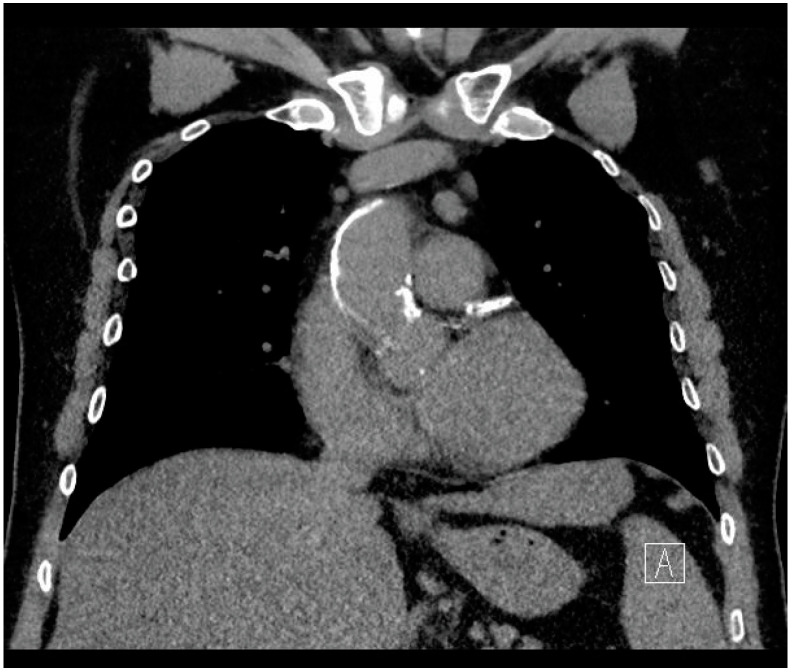
An example of massive calcifications detected by preoperative CT screening.

In the present study, we decided to evaluate the influence of standardized CT screening in a cohort of patients with cardiovascular disease and a high risk of calcification, with old age, chronic renal dialysis, peripheral vascular disease, or carotid stenosis, as atherosclerotic diseases of the vascular system have been recognized as independent risk factors for stroke in previous studies [28,29]. A statistical analysis showed that the implementation of preoperative CT screening in this patient cohort was associated with lower postoperative stroke rates. An analysis of the patient data confirmed that multiple patients were preoperatively identified as having massive calcifications, and the surgical strategy was changed to address this finding, as shown in Figure 2. Furthermore, in our experience, the identification of even minor calcifications alerted the surgical team to optimize the aortic cannulation and clamping sites, and to use epi-aortic ultrasound more frequently to study these sites meticulously to exclude soft plaque and calcifications. Nevertheless, the logistic regression was able to predict the safety from stroke risk much higher than its capability of predicting stroke. This should be taken into consideration and regard the presented model as an assurance of safety in patients where no calcifications are detected rather than confirmation of risk in the other patients. Furthermore, this confirms the need of larger studies to improve the model and increase the capability to predict a stroke.

An analysis of the risk factors associated with higher stroke rates revealed age, in addition to a lack of CT screening, as a preoperative risk factor. However, hypertension and smoking were not identified as risk factors in the present study. This could be explained by the fact that approximately 90% of the patients in both groups were hypertensive, and none were smokers, which renders the study insufficiently powered to confirm or refute the data of previous studies in this regard.

Further analyses showed that aortic surgery, aortic valve surgery, and rethoracotomy were associated with higher stroke rates. These findings are consistent with previously published data, confirming that aortic surgery is an independent risk factor for postoperative stroke [29]. The report by Anyanwu et al. did not include aortic valve surgery or rethoracotomy as independent risk factors. However, Messé et al. reported a 17% stroke rate in patients who underwent surgical aortic valve replacement in a prospective cohort study in high-volume centers with extensive expertise [2]. These data suggest that aortic valve replacement is associated with higher postoperative stroke rates, but this requires further investigation. The higher stroke rate in patients who underwent rethoracotomy might be due to the frequent hemodynamic instability in this patient cohort, with possible hypotensive phases with cerebral malperfusion, or it might be due to its higher correlation with major aortic surgery. As multivariate analysis could not build a robust regression model, the independent effect of rethoracotomy could not be confirmed or refuted.

This study had a few limitations. First, it was a single-center study. Furthermore, as mentioned, no robust regression model could be built to reveal the independent risk factors affecting the postoperative stroke rate. The weakness of the regression model might be largely attributed to the small cohort size and the multifactorial nature of postoperative stroke. Nevertheless, the identification of risk factors for postoperative stroke was beyond the objective of this study, which was to investigate the effect of preoperative CT screening on postoperative stroke.

## 5. Conclusions

Preoperative non-contrast CT screening decreases the postoperative stroke rate in patients at high risk of aortic calcification who are undergoing cardiac surgery. The preoperative detection of aortic calcification helped optimize the surgical approach. In patients aged >75 years, or with a history of peripheral vascular disease, carotid stenosis, or dialysis, preoperative non-contrast chest computed tomography should be performed whenever possible. Further evaluation is necessary to confirm the data in larger cohorts and to determine the risk factors that should be considered for patient inclusion in the screening process.

## Figures and Tables

**Table 1:** Demographic and preoperative data.

	CT Group *n* = 129	Control Group *n* = 261	*p*-Value
Age	75.2 ± 6.8	76.6 ± 7.0	0.061
Diabetes	29.5%	39.8%	0.057
Arterial hypertension	84.5%	90.1%	0.093
Pulmonary hypertension	14.7%	15.7%	0.882
NYHA III & IV	79.1%	71.6%	0.174
Redo procedure	7.0%	8.8%	0.695
Recent infarction	3.1%	0.4%	0.043
Cardiogenic shock	0.8%	0.0%	0.383
Recent resuscitation	0.8%	0.0%	0.282
Atrial fibrillation	20.2%	16.7%	0.729
LVEF	55.1 ± 11.1	51.9 ± 14.4	0.023
Coronary artery disease	82.2%	85.7%	0.374
Peripheral vascular disease	22.5%	19.2%	0.451
Carotid stenosis	18.6%	28.0%	0.047
Aortic aneurysm	4.7%	6.5%	0.648
Creatinine level	1.10 ± 0.40	1.18 ± 0.50	0.083
Chronic dialysis	1.6%	0.8%	0.362
COPD, %	16.3	14.2	0.822
History of CVI, %	14.0	17.9	0.616

Abbreviations: n, number of patients; NYHA: New York Heart Association; LVEF: left ventricular ejection fraction; COPD: chronic obstructive pulmonary disease; CVI: cerebrovascular incidence.

**Table 2:** Intraoperative procedures.

Variable	Group 1	Group 2	*p*-Value
Urgent surgery	37.8%	44.8%	0.277
Operative time (min)	268 ± 84	248 ± 95	0.046
CPB time	121 ± 67	107 ± 58	0.032
Aortic cross-clamp time	80 ± 49	75 ± 39	0.244
Circulatory arrest time	31 ± 21	37 ± 25	0.744
Median Sternotomy	82.2%	87.1%	0.305
Partial upper mini-sternotomy	3.9%	2.3%	0.305
Anterolateral thoracotomy	10.1%	6.8%	0.305
Other (e.g., inferior mini-sternotomy)	3.8%	3.8%	0.305
Mitral valve surgery	22.5%	17.8%	0.392
Aortic valve surgery	26.4%	24.5%	0.710
Tricuspid valve surgery	9.3%	6.6	0.413
Aortic surgery	6.2%	5.0%	0.639
Coronary surgery	72.9%	76.7%	0.452
Minimally invasive surgery	16.3%	10.1%	0.098
Closure of left atrial appendage	10.1%	10.4%	1.000
Carotid endarterectomy	1.6%	6.2%	0.042
Atrial ablation	9.3%	8.9%	1.000
Other procedures	7.8%	4.7%	0.247
Combined procedures	36.4%	34.1%	0.653

Abbreviations: min.: minutes; CPB: cardiopulmonary bypass.

**Table 3:** Postoperative data.

Outcome	Group 1	Group 2	*p*-Value
Postoperative stroke	3.1%	8.4%	0.047
Postoperative delirium	24.0%	20.2%	0.651
Reintubation	0.8%	2.3%	0.296
Hospital stay (days)	15 ± 14	15 ± 18	0.440
Low cardiac output	0.8%	0.0%	0.329
Rethoracotomy	7.0%	8.7%	0.548
Pacemaker implantation	3.9%	2.3%	0.369
Renal replacement therapy	5.4%	5.7%	0.197
30-day mortality	4.7%	4.9%	0.899

**Table 4:** Univariate analysis of factors affecting postoperative stroke.

Variable	Significance
CT screening	0.035
Age	0.028
Aortic valve surgery	0.017
Aortic surgery	0.009
Rethoracotomy	0.013

**Table 5:** Multivariate analysis of independent factors affecting postoperative stroke.

	Regression Coefficient	Standard Error	Wald	df	Sig.	Exp
Age	0.087	0.042	4.289	1	**0.038**	1.091
Male sex	−0.409	0.508	0.650	1	0.420	0.664
BMI	−0.040	0.055	0.518	1	0.472	0.961
CT screening	1.408	0.657	4.591	1	**0.032**	4.089
Aortic valve surgery	0.964	0.471	4.184	1	**0.041**	2.621
Aortic surgery	2.987	0.822	13.188	1	**<0.001**	19.817
Coronary surgery	0.895	0.604	2.192	1	0.139	2.447
Rethoracotomy	−1.257	0.581	4.686	1	**0.030**	0.284
Aortic aneurysm	2.090	1.289	2.629	1	0.105	8.082
Constant	−11.488	4.120	7.773	1	0.005	0.000

The bolded numbers are the significant variables.

## Data Availability

The data presented in this study are available on request from the corresponding author due to privacy and ethical restrictions. The date will be curated for 5 years after publication.
